# Genomic and Biotechnological Characterization of the Heavy-Metal Resistant, Arsenic-Oxidizing Bacterium *Ensifer* sp. M14

**DOI:** 10.3390/genes9080379

**Published:** 2018-07-27

**Authors:** George C diCenzo, Klaudia Debiec, Jan Krzysztoforski, Witold Uhrynowski, Alessio Mengoni, Camilla Fagorzi, Adrian Gorecki, Lukasz Dziewit, Tomasz Bajda, Grzegorz Rzepa, Lukasz Drewniak

**Affiliations:** 1 Laboratory of Microbial Genetics, Department of Biology, University of Florence, via Madonna del Piano 6, 50019 Sesto Fiorentino, Italy; alessio.mengoni@unifi.it (A.M.); camilla.fagorzi@unifi.it (C.F.); 2 Laboratory of Environmental Pollution Analysis, Faculty of Biology, University of Warsaw, Miecznikowa 1, 02-096 Warsaw, Poland; w.uhrynowski@biol.uw.edu.pl (W.U.); ldrewniak@biol.uw.edu.pl (L.Dr.); 3 Faculty of Chemical and Process Engineering, Warsaw University of Technology, Warynskiego 1, 00-645 Warsaw, Poland; jan.krzysztoforski@pw.edu.pl; 4 Department of Bacterial Genetics, Institute of Microbiology, Faculty of Biology, University of Warsaw, Miecznikowa 1, 02-096 Warsaw, Poland; agorecki@biol.uw.edu.pl (A.G.); ldziewit@biol.uw.edu.pl (L.Dz.); 5 Department of Mineralogy, Petrography and Geochemistry, Faculty of Geology, Geophysics and Environmental Protection, AGH University of Science and Technology, Mickiewicza 30, 30-059 Krakow, Poland; bajda@agh.edu.pl (T.B.); gprzepa@cyf-kr.edu.pl (G.R.)

**Keywords:** *Ensifer* (*Sinorhizobium*) sp. M14, arsenic-oxidizing bacteria, heavy metal resistance, draft genome sequence, comparative genomic analysis, biosafety, biotechnology for arsenic removal, adsorption, water treatment, in situ (bio)remediation

## Abstract

*Ensifer* (*Sinorhizobium*) sp. M14 is an efficient arsenic-oxidizing bacterium (AOB) that displays high resistance to numerous metals and various stressors. Here, we report the draft genome sequence and genome-guided characterization of *Ensifer* sp. M14, and we describe a pilot-scale installation applying the M14 strain for remediation of arsenic-contaminated waters. The M14 genome contains 6874 protein coding sequences, including hundreds not found in related strains. Nearly all unique genes that are associated with metal resistance and arsenic oxidation are localized within the pSinA and pSinB megaplasmids. Comparative genomics revealed that multiple copies of high-affinity phosphate transport systems are common in AOBs, possibly as an As-resistance mechanism. Genome and antibiotic sensitivity analyses further suggested that the use of *Ensifer* sp. M14 in biotechnology does not pose serious biosafety risks. Therefore, a novel two-stage installation for remediation of arsenic-contaminated waters was developed. It consists of a microbiological module, where M14 oxidizes As(III) to As(V) ion, followed by an adsorption module for As(V) removal using granulated bog iron ores. During a 40-day pilot-scale test in an abandoned gold mine in Zloty Stok (Poland), water leaving the microbiological module generally contained trace amounts of As(III), and dramatic decreases in total arsenic concentrations were observed after passage through the adsorption module. These results demonstrate the usefulness of *Ensifer* sp. M14 in arsenic removal performed in environmental settings.

## 1. Introduction

The development and implementation of bioremediation technologies based on bioaugmentation requires the selection of appropriate microbial strains. A basic requirement of strains used as bioaugmentation agents is their ability to survive in the environment into which they are introduced. Thus, such strains are usually characterized by high tolerance to heavy metals [1,2], resistance and ability to use organic (sometimes toxic) compounds [3,4], resistance to antibiotics [5], and an ability to thrive in the presence of local bacteriophages and microorganisms. Another important feature of strains used in bioaugmentation is their ability to perform effective transformation of the particular compound under changing environmental conditions (e.g., temperature, humidity, and pH). This is always the critical limitation, as many strains effective under laboratory conditions are, in fact, ineffective in field applications. Microorganisms suitable in bioremediation should maintain their activity in various seasons and under variable substrate inflow. A very important factor influencing the decision to apply a given microorganism in practice is also its interaction with the environment [6]. Strains that contribute to the uncontrolled release of contaminants, dissemination of antibiotic resistance genes, or disrupt the functioning of the ecosystem (e.g., by eliminating key microorganisms) should not be applied in open (uncontrolled) usage.

In this study, we provide a detailed characterization of *Sinorhizobium* sp. M14 (renamed here to *Ensifer* sp. M14 due to its phylogenetic positioning within the *Ensifer* clade), which is a strain with high potential to be used in bioremediation technologies for the removal of arsenic from contaminated waters and wastewaters. *Ensifer* sp. M14 is a psychrotolerant strain that was isolated from the microbial mats present in the arsenic-rich bottom sediments of an abandoned gold mine in Zloty Stok (Poland) [7]. The arsenic concentration in the mine waters reaches ~6 mg L^−1^, while in the microbial mats the level of accumulated arsenic is close to 20 g L^−1^ [8]. Previous physiological studies showed that *Ensifer* sp. M14 tolerates extremely high concentrations of arsenate [As(V)—up to 250 mM] and arsenite [As(III)—up to 20 mM], and is able to oxidize As(III) both chemolithoautotrophically [using arsenite or arsenopyrite (FeAsS) as a source of energy] and heterotrophically [7]. Batch experiments performed under various conditions of pH, temperature, and arsenic concentration confirmed the high adaptive potential of *Ensifer* sp. M14 [9]. The strain was capable of intensive growth and efficient biooxidation in a wide range of conditions, including low temperature [As(III) oxidation rate = 0.533 mg L^−1^ h^−1^ at 10 °C]. Continuous flow experiments under environment-like conditions (2 L flow bioreactor) showed that *Ensifer* sp. M14 efficiently transforms As(III) into As(V) [24 h of residence time was sufficient to oxidize 5 mg L^−1^ of As(III)], but its activity depended mainly on the retention time in the bioreactor, which may be accelerated by stimulation with yeast extract as a source of nutrients [9].

Analysis of the extrachromosomal replicons of *Ensifer* sp. M14 revealed that its arsenic metabolism properties are linked with the presence of the mega-sized plasmid pSinA (109 kbp) [10]. The loss of the pSinA plasmid from *Ensifer* sp. M14 cells (using a target-oriented replicon curing technique [11]) eliminated the ability to oxidize As(III), and caused deficiencies in resistance to arsenic and heavy metals (Cd, Co, Zn, and Hg). In turn, the introduction of this plasmid into other representatives of the *Alphaproteobacteria* showed that cells with pSinA acquired the ability to oxidize arsenite and exhibited higher tolerance to arsenite than their parental, pSinA-less, wild-type strains. Horizontal transfer of arsenic metabolism genes by *Ensifer* sp. M14 was also confirmed in microcosm experiments [10]. The plasmid pSinA was successfully transferred via conjugation into indigenous bacteria of *Alpha*- and *Gammaproteobacteria* classes from the microbial community of As-contaminated soils. Transconjugants carrying plasmid pSinA expressed arsenite oxidase and stably maintained pSinA in their cells after approximately 60 generations of growth under nonselective conditions [10].

The second mega-sized replicon of *Ensifer* sp. M14—plasmid pSinB (300 kbp)—also plays an important role in the adaptation of the host to the mine environment. Structural and functional analysis of this plasmid showed that it carries gene clusters involved in heavy metals resistance. Among these are genes encoding efflux pumps, permeases, transporters, and copper oxidases, which are responsible for resistance to arsenic, cobalt, zinc, cadmium, iron, mercury, nickel, copper, and silver [12].

In this paper, we obtained a draft genomic sequence of *Ensifer* sp. M14 and performed complex genome-guided characterization of this bacterium. Special considerations were given to (i) determination of the metabolism of phosphate, sulfur, iron, and one-carbon substrates, and (ii) investigation of the biosafety of *Ensifer* sp. M14 in the context of its release to the environment (e.g., determination of the presence of virulence and antibiotic resistance genes). These analyses revealed hints about the potential application of this strain in biotechnological applications; for example, the ability of it to survive environmental stresses, and whether it is likely to pose a safety risk. As the genomic analyses were consistent with *Ensifer* sp. M14 having potential application in biotechnology, we performed a large-scale simulation of the usage of M14 in the biological and chemical removal of arsenic from contaminated waters. The results support that the developed low-cost approach is an efficient method for the removal of arsenic from contaminated water.

## 2. Materials and Methods

### 2.1. Genome Sequencing, Assembly, and Annotation

*Ensifer* sp. M14 (available on request from the authors) was grown at 30 °C to stationary phase in TY medium (5 g L^−1^ tryptone, 3 g L^−1^ yeast extract, and 0.4 g L^−1^ calcium chloride). Genomic DNA was isolated from the culture using a cetyltrimethylammonium bromide (CTAB) method [13] modified for bacterial DNA isolation as described by the Joint Genome Institute [14]. Sequencing was performed at IGATech (Udine, Italy) using an Illumina HiSeq2500 instrument with 125-bp paired-end reads. Two independent sequencing runs were performed. Reads were assembled into scaffolds using SPAdes v3.9.0 [15,16]. The scaffolds returned by SPAdes were parsed to remove those with less than 10× coverage or with a length below 200 nucleotides. Using FastANI [17], one-way average nucleotide identity (ANI) of the *Ensifer* sp. M14 assembly was calculated against the 887 alpha-proteobacterial genomes available through the National Center for Biotechnological Information (NCBI) with an assembly level of ‘complete’ or ‘chromosome’. The 10 genomes most closely related to *Ensifer* sp. M14 were identified on the basis of the ANI results. These 10 genomes, together with the complete pSinA and pSinB plasmid sequences [10,12], were used as reference genomes for further scaffolding of the assembly using MeDuSa [18]. The *Ensifer* sp. M14 assembly was then annotated using prokka version 1.12-beta [19], annotating coding regions with Prodigal [20], tRNA with Aragon [21], rRNA with Barrnap (github.com/tseemann/barrnap), and ncRNA with Infernal [22] and Rfam [23]. The predicted coding sequences were associated with Cluster of Orthologous Genes (COG) categories, Gene Ontology (GO) terms, Kyoto Encyclopedia of Genes and Genomes (KEGG) pathway terms, and eggNOG annotations using eggNOG-mapper version 0.99.2-3-g41823b2 [24]. The assembly was deposited to NCBI with the GenBank accession QJNR00000000 (the version described in this paper is version QJNR01000000) and the BioSample accession SAMN09254189.

### 2.2. Phylogenetic Analysis

Initially, all 133 *Sinorhizobium*/*Ensifer* genomes available through NCBI, regardless of assembly level, were downloaded. FastANI [17] was used to calculate one-way ANI values between *Ensifer* sp. M14 and each of the 133 downloaded genomes. Only the strains meeting at least one of the following two requirements were kept for further analyses: (i) had a genome assembly level of ‘complete’ or ‘chromosome’, or (ii) had an ANI value of at least 85% compared to *Ensifer* sp. M14. This resulted in a final set of 46 strains, when including *Ensifer* sp. M14.

The pangenome of the 46 strains was calculated using Roary version 3.11.3 [25], as described below, following re-annotation with prokka version 1.12-beta [19]. Included in the Roary output was a concatenated nucleotide alignment of the 1652 core genes, each individually aligned with PRANK [26]. The core gene alignment was used to build a maximum likelihood phylogeny with RAxML version 8.2.9 [27] using the following command:

raxmlHPC-HYBRID-SSE3–T 5–s input.fasta–N autoMRE–n output–f a–p 12345–x 12345–m GTRCAT.

The final tree is the bootstrap best tree following 50 bootstrap replicates, and was visualized using the online iTOL (Interactive Tree of Life) webserver [28].

Strains were grouped into putative species on the basis of ANI and average amino acid identity (AAI) values, using thresholds of 96% for both measures. Groupings for ANI were the same at thresholds of 96% and 94%. Pairwise ANI values were calculated between each strain using FastANI [17], and the values in both directions were averaged. The CompareM workflow (github.com/dparks1134/CompareM) was used for calculating the AAI values. In the CompareM workflow, orthologous proteins were first identified using DIAMOND with the sensitive setting [29], and thresholds of 40% identity over 70% the length of the protein and a maximum e-value of 1e^−12^ were applied, as these are the thresholds used in the myTaxa program [30].

### 2.3. Sinorhizobium/Ensifer Pangenome Calculation

All 46 strains included in the phylogenetic analyses were reannotated using prokka version 1.12-beta [19], to ensure consistent annotation. The pangenome of the 46 reannotated strains was then determined with Roary version 3.11.3 [25], using an amino acid identity threshold of 80% and the following command:

roary–p 20–f Output–e–I 80–g 100,000 Input/*.gff.

For comparison of the gene content of *Ensifer* sp. M14, *Ensifer* sp. A49, *Ensifer adhaerens* OV14, and *Ensifer adhaerens* Casida A, the data was extracted from the full 46-strain pangenome. The complete gene presence/absence output from Roary is provided as Data Set S1. Several short proteins of *Ensifer* sp. M14 were not present in the output of the Roary analysis; these proteins were not considered when identifying unique genes.

### 2.4. Comparative Genomics of Arsenic Oxidizing Bacteria

The genomes of *Agrobacterium tumefaciens* 5A [31], *Agrobacterium tumefaciens* Ach5 [32], *Ensifer adhaerens* OV14 [33], *Neorhizobium galegae* HAMBI 540 [34], and *Rhizobium* sp. NT-26 [35] were downloaded from NCBI GenBank and reannotated using prokka, as described above for *Ensifer* sp. M14. The GenBank files of the re-annotated genomes, and the *Ensifer* sp. M14 genome, were uploaded to the KBase webserver [36], and OrthoMCL [37] was run on the KBase server using an e-value threshold of 1e^−12^. Identification of phosphate transport and arsenic resistance genes in other bacterial genomes (*Achromobacter arsenitoxydans* SY8 [38], *Herminiimonas arsenicoxydans* ULPAs1 [39], and *Pseudomonas stutzeri* TS44 [40]) was accomplished by manually searching the GenBank file of the RefSeq annotated genomes [41].

### 2.5. Identification of Prophage Loci

PhiSpy version 3.2 [42], implemented in Python, was used to predict phage genes. The *Ensifer* sp. M14 GenBank file produced with prokka was converted to SEED format using the genbank_to_seed.py script. The converted file was then used as input for the PhiSpy.py script, using the generic test set for training.

### 2.6. Identification of Putative Antibiotic Resistance Genes

To identify putative antibiotic resistance genes, the Resistance Gene Identifier (RGI) in the Comprehensive Antibiotic Resistance Database (CARD) software was used [43]. Hits showing at least 50% identity with the reference protein were considered significant. Each hit was verified manually using BLASTp analysis.

### 2.7. Analysis of the Antimicrobial Susceptibility Patterns

To determine the antimicrobial susceptibility patterns of *Ensifer* sp. M14, minimum inhibitory concentrations (MICs) of 11 antimicrobial agents were assessed using Etest™ (Liofilchem, Roseto degli Abruzzi, Italy). The analysis was conducted according to the European Committee on Antimicrobial Susceptibility Testing (EUCAST) recommendations [44]. The following antibiotics (selected based on the bioinformatic analyses that identified putative antibiotic resistance genes) were used: (i) aminoglycosides–gentamicin (GN; concentration of antibiotic: 0.064–1024 μg mL^−1^ Roseto degli Abruzzi^1^); (ii) β-lactams (penicillin derivatives)–ampicillin (AMP; 0.016–256 μg mL^−1^); (iii) β-lactams (cephalosporins)–cefixime (CFM; 0.016–256 μg mL^−1^); (iv) β-lactams (cephalosporins)–cefotaxime (CTX; 0.016–256 μg mL^−1^); (v) β-lactams (cephalosporins)–ceftriaxone (CRO; 0.016–256 μg mL^−1^); (vi) fluroquinolones–ciprofloxacin (CIP; 0.002–32 μg mL^−1^); (vii) fluroquinolones–moxifloxacin (MXF; 0.002–32 μg mL^−1^); (viii) phenicols–chloramphenicol (C; 0.016–256 μg mL^−1^); (ix) rifamicyns–rifampicin (RD; 0.016–256 μg mL^−1^); (x) sulfonamides–trimethoprim (TM; 0.002–32 μg mL^−1^); and (xi) tetracyclines–tetracycline (TE; 0.016–256 μg mL^−1^). The susceptibility testing was performed at 30 °C for 20 h. After incubation, plates were photographed and MICs were defined. Antimicrobial susceptibility data were interpreted according to the EUCAST breakpoint table version 8.0 [45].

### 2.8. Search for Symbiotic Proteins

A custom pipeline based on the use of hidden Markov models (HMM) was used to search the proteomes of all 46 *Sinorhizobium*/*Ensifer* strains for the presence of the nodulation proteins NodA, NodB, and NodC, as well as for the nitrogenase proteins NifH, NifD, and NifK. This pipeline is dependent on HMMER version 3.1b2 [46], and the complete Pfam-A version 31.0 (16,712 HMMs) and TIGERFAM version 15.0 (4488 HMMs) databases [47,48]. After downloading the HMM databases, hmmconvert was used to ensure consistent formatting. The two databases were combined into a single HMM database, and then converted into a searchable database with hmmpress. Additionally, the HMM seed alignments for NodA (TIGR04245), NodB (TIGR04243), NodC (TIGR04242), NifH (TIGR01287), NifD (TIGR01282), and NifK (TIGR01286) were downloaded from the TIGRFAM database [47].

For each HMM seed alignment, a HMM was built using hmmbuild, and the output was then searched against the complete set of *Sinorhizobium*/*Ensifer* proteins using hmmsearch. The output was parsed, and the amino acid sequences for each of the hits (regardless of e-value) were collected. Each set of sequences were then searched against the combined HMM database using hmmscan, and the output parsed to identify the top scoring HMM hit for each query protein. Proteins were annotated as follows: NodA if the top hit was TIGR04245 (TIGRFAM) or NodA (Pfam); NodB if the top hit was TIGR04243 (TIGRFAM); NodC if the top hit was TIGR04242 (TIGRFAM); NifH if the top hit was TIGR01287 (TIGRFAM) or Fer4_NifH (Pfam); NifD if the top hit was TIGR01282 (TIGRFAM), TIGR01860 (TIGRFAM), or TIGR01861 (TIGRFAM); NifK if the top hit was TIGR02932 (TIGRFAM), TIGR02931 (TIGRFAM), or TIGR01286 (TIGRFAM).

### 2.9. Cluster of Orthologous Genes Functional Annotation

Proteomes were annotated with COG functional categories using eggNOG-mapper version 0.99.2-3-g41823b2 [24]. The output of eggNOG-mapper was parsed with a custom Perl script to count the percentage of proteins annotated with each functional category. Fisher exact tests, performed using MATLAB R2016b (www.mathworks.com), were performed to identify statistically significant differences (*p* < 0.05) between *Ensifer* sp. M14 and the other strains.

### 2.10. In Silico Metabolic Reconstruction and Constraint-Based Modelling

Metabolic reconstruction steps and constraint-based metabolic modeling were performed in MATLAB 2017a (Mathworks, Natick, MA, USA), using the Gurobi 7.0.2 solver (gurobi.com), SBMLToolbox 4.1.0 [49], libSBML 5.13.0 [50], and scripts from the COBRA Toolbox [51] and the Tn-Core Toolbox [52]. The ability of the *Ensifer* sp. M14 model to grow when individually provided with 163 carbon sources was tested using flux balance analysis (FBA) as implemented in the ‘optimizeCbModel’ function of the COBRA Toolbox.

An initial draft metabolic reconstruction was prepared using the online KBase webserver [36]. The *Ensifer* sp. M14 genome was uploaded and re-annotated with RAST functions using the ‘annotate microbial genome’ function. The re-annotated genome was used to build a draft model with the ‘build metabolic model’ function, performing gap-filling on a glucose minimal medium, and with automatic biomass template selection. This reconstruction was downloaded in SBML format, and then imported into MATLAB as a COBRA formatted metabolic model for further manipulation. After removing duplicate genes from the gene list and updating the gene-reaction rules appropriately, the model was expanded based on the reaction content of the curated iGD1575 and iGD726 metabolic reconstructions of the closely related species *Sinorhizobium meliloti* [53,54]. First, a BLAST bidirectional best hit approach was used to identify putative orthologs (at least 70% identity over at least 70% the protein length) between *S. meliloti* Rm1021 and *Ensifer* sp. M14. All *S. meliloti* genes without a putative ortholog in *Ensifer* sp. M14 were deleted from the iGD1575 and iGD726 models, and the constrained reactions removed. Next, the reactions of iGD726 and the draft *Ensifer* sp. M14 model were compared based on their equations, and all reactions unique to iGD726 were identified and transferred to the *Ensifer* sp. M14 model. Exceptions were iGD726 reactions that differed from a reaction in the *Ensifer* sp. M14 model only in the presence/absence of a proton or in metabolite stoichiometry. This process was then repeated, transferring the unique reactions of iGD1575 to the partially expanded model. When transferring reactions, associated genes were also transferred and changed to the name of the *Ensifer* sp. M14 orthologs. Following the expansions, all reactions producing dead-end metabolites were iteratively removed from the model. The final model contained 1491 genes, 1561 reactions, and 1105 metabolites, and is available in Data Set S2.

### 2.11. Prediction of Secondary Metabolism

Loci encoding secondary metabolic pathways were predicted in the *Ensifer* sp. M14 genome using the antiSMASH webserver [55]. The *Ensifer* sp. M14 GenBank file was uploaded to the bacterial version of antiSMASH, and the analysis was run with all options selected with default parameters.

### 2.12. Construction of a Pilot-Scale Installation for Arsenic Bioremediation

**Figure 1:**
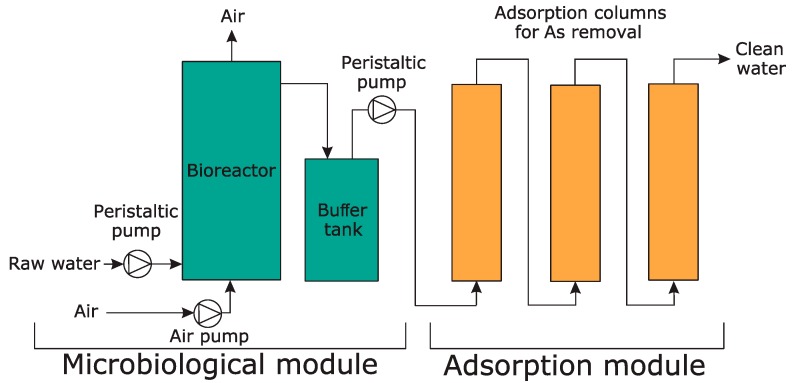
The pilot-scale installation used for remediation of arsenic contaminated water. The image is a schematic representation of the pilot-scale installation developed as part of this work. Both the microbiological and adsorption modules are shown.

A pilot-scale installation for the removal of arsenic from contaminated waters was developed. The installation was operated using water from a dewatering system of a former gold mine located in the Zloty Stok area (SW Poland), which is highly polluted with arsenic. The total arsenic concentration, arsenic speciation, as well as detailed chemical and physical characteristics of the water are presented elsewhere [56]. The installation consisted of two modules: the microbiological module and the adsorption module (Figure 1).

The microbiological module was based on the activity of *Ensifer* sp. M14, which was used as an arsenite biooxidizer. This module included a 200 L bioreactor with an electric heater. The contaminated water flowing out from the gold mine was fed into the bioreactor through a pressure reducer and a peristaltic pump at a volume flow rate of 8.33 L h^−1^, corresponding to a residence time of 24 h in the bioreactor. Outflow of the water occurred as overflow in the upper part of the bioreactor. To increase the effectiveness of the arsenite biooxidition, the bioreactor was equipped with an additional aeration system that consisted of an air pump producing compressed air. The additional aeration system was included in our previous study and showed that the arsenite oxidation efficiency of *Ensifer* sp. M14 is higher in the presence of additional aeration during continuous culturing [9]. Moreover, yeast extract was added to the bioreactor as a source of vitamins (growth supplements). Fifty grams of powdered yeast extract (Sigma-Aldrich, St. Louis, MO, USA) was added to the bioreactor twice a week. This was done as we previously observed that the presence of yeast extract led to an increase in the growth and efficiency of arsenite biooxidation of *Ensifer* sp. M14 during continuous culturing [9]. This relationship was also confirmed in other papers [10,57]. The supply of air also contributed to the mixing of the bioreactor content. The bioreactor was equipped with a multifunctional electrode dedicated to controlling the chemical and physical parameters of water, specifically, to monitor pH, redox potential, and temperature (Hydrolab HL4, OTT Hydromet, Kempten, Germany). The water leaving the bioreactor was fed into a 60 L buffer tank, which functioned as the connecting element between the bioreactor and the adsorption module. The inclusion of the buffer tank helped maintain a constant water level in the adsorption columns and ensured a constant flow of water from the bioreactor to the adsorption columns.

The adsorption module consisted of three columns (17 L volume each) filled with granulated bog iron ores (about 15 kg per column) and connected in series (Figure 1). The detailed chemical and physical parameters, chemical composition, and stability of the adsorbent were presented previously [56]. Contaminated water from the buffer tank (after passing through the microbiological module) was fed into the first column using a second peristaltic pump at a volume flow rate of 8.33 L h^−1^, which corresponded to approximately one hour of residence time per column.

The installation was also equipped with a process control system (operated at the location of the pilot plant or remotely via a Global System for Mobile Communications (GSM)) that monitored and controlled key process parameters including the volume flow rate of the water, the water temperature at the inlet, in the bioreactor, and at the outlet of the pilot plant, as well as the ambient temperature.

### 2.13. Installation Start-Up

Scale-up of the installation (from laboratory scale to pilot scale) required the development of procedures for successful start-up based on the results of our previous study [9]. The first step of the start-up of the microbiological module was inoculation of the bioreactor with an appropriate amount of *Ensifer* sp. M14. The bioreactor filled with arsenic contaminated water was inoculated with 200 mL of a highly concentrated overnight culture of *Ensifer* sp. M14 suspended in 0.85% NaCl solution. The initial OD_600_ in the bioreactor was 0.01. In earlier experiments, it was determined that a starting cell density of 10^8^ CFU mL^−1^ (which corresponds to an OD_600_ of 0.1) is required for the installation to work properly [9]. To increase the density of the *Ensifer* sp. M14, the water in the bioreactor was supplemented with powdered yeast extract to a final concentration of 0.04%. Additionally, aeration was applied. Finally, the temperature of the water was increased (from 10 to 22 °C) with the use of an electric heater placed in the bioreactor. Application of all these treatments led to an OD_600_ value of 0.1 within 24 h.

Start-up procedures related to the adsorption module mainly concerned the preparation of the adsorbent for its usage. After filling the columns with granulated bog iron ores, it was necessary to condition the adsorbent (rinsing the adsorbent with the tap water without arsenic) to remove all the loosely bound fractions.

### 2.14. Biological and Chemical Analyses

Arsenic speciation was investigated with the use of ion chromatography on an IonPac AS18 (2 mm, Dionex, Lübeck, Germany) column on an ICS Dionex 3000 (Lübeck, Germany) instrument equipped with an ASRS^®^ 2 mm suppressor, which was coupled to a ZQ 2000 mass spectrometer via an electrospray source (Waters, Milford, MA, USA) according to the method described by Debiec et al. [9]. In the adsorption module, the total arsenic concentration was investigated. Total arsenic concentration was measured using a Graphite Furnace Atomic Absorption Spectrometry (GFAAS; AA Solaar M6 Spectrometer, TJA Solutions, Waltham, MA, USA). Arsenic standard solutions (Merck, Darmstadt, Germany) were prepared in 3% HNO_3_. The pH and redox potential were measured only in the microbiological module. Samples of raw water, water from the bioreactor, as well as water at the inflow and outflow of each adsorption column were collected once a day during the first 8 days, and then three times a week up to day 40. Samples taken from the bioreactor were stored at −20 °C, while samples collected from the adsorption module were stored at 4 °C. This experiment was repeated twice.

## 3. Results and Discussion

### 3.1. Sequencing of the Ensifer sp. M14 Genome

The draft genome sequence of *Ensifer* sp. M14 was obtained as described in the Materials and Methods, and the general genomic features are described in Table 1.

The assembly consists of 7,345,249 bp spread over 45 scaffolds at an average coverage of 118×. Of the 45 scaffolds, 12 are over 40 kbp in size and account for 98.7% of the assembly. Based on similarity searches of the scaffolds, previous plasmid profiling of *Ensifer* sp. M14 [10,12], and the finished genomes of related strains [33,58], we predict that the *Ensifer* sp. M14 genome consists of one chromosome (at least 4.4 Mbp in size), two additional large replicons (chromids and/or large megaplasmids, at least 1.6 Mbp and 0.6 Mbp in size), and the two previously reported smaller megaplasmids (pSinA and pSinB, 109 kbp and 300 kbp, respectively, based on previous papers [10,12]). A total of 6874 coding sequences were predicted, which is more than the 6218 predicted in *S. meliloti* Rm1021 and the 6641 of *E. adhaerens* Casida A, but less than the 7033 predicted in *E. adhaerens* OV14 [33,58,59]. Six putative prophages were identified on Scaffold 4 (the chromosome) using PhiSpy [42]; these ranged in size from 21 to 65 genes, and accounted for a total of 292 genes (Data Set S3). However, no CRISPR loci were detected during annotation with prokka [19]; a questionable, short CRIPSR with one spacer was detected with CRISPRfinder [59], but its location within a predicted coding region suggests it is unlikely to be a true CRISPR locus. No evidence for the presence of the common nodulation genes *nodABC* or the nitrogenase genes *nifHDK* was found using a hidden Markov model based approach. The *Ensifer* sp. M14 assembly has been deposited in GenBank under the accession QJNR00000000, as part of the BioSample SAMN09254189.

### 3.2. Taxonomic Analysis of Ensifer sp. M14

**Figure 2:**
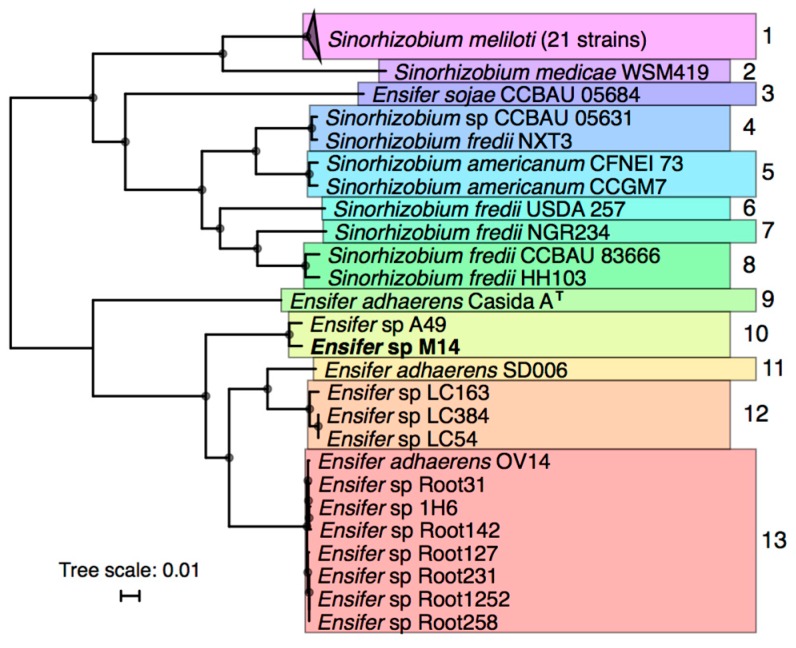
Phylogeny of a selected 46 *Sinorhizobium*/*Ensifer* strains with a publicly available whole genome sequence. An unrooted RAxML maximum likelihood phylogeny of 46 *Sinorhizobium*/*Ensifer* strains was prepared on the basis of the concatenated nucleotide alignments of 1652 core genes. The presented tree is the bootstrap best tree following 50 bootstrap replicates, and the scale represents the mean number of nucleotide substitutions per site. Nodes with 100% bootstrap support are indicated by the black circles. The colors and numbers to the right of the tree are used to indicate strains that group into putative species on the basis of average nucleotide identity (>96% ANI; same results were obtained with >94% ANI) and average amino acid identity (>96% AAI), as described in the Materials and Methods. Type strains are indicated by the ‘T’. The accessions for all strains included in this figure are provided in Table S1.

Phylogenetic analyses were performed to identify the relationships between *Ensifer* sp. M14 and previously sequenced *Sinorhizobium*/*Ensifer* strains. Forty-five *Sinorhizobium*/*Ensifer* genomes were downloaded from the NCBI database (see Materials and Methods for criteria for strain inclusion), and a maximum likelihood phylogeny of these strains plus *Ensifer* sp. M14 was built based on 1652 core genes (Figure 2).

The 46 strains were grouped into putative species on the basis of whole genome ANI and AAI values (Figures S1 and S2). The results revealed that *Ensifer* sp. M14 is closely related to *Ensifer* (*Sinorhizobium*) sp. A49 (98.5% ANI and 98.9% AAI), and that these strains likely belong to a new species. *Ensifer* sp. A49 was previously isolated from soil of the Fureneset Rural Development Centre of Fjaler, Norway [60]. However, the pSinA and pSinB plasmids, carrying genes involved in arsenic oxidation and heavy metal resistance [10,12], appear to be specific to *Ensifer* sp. M14 and may therefore have been gained during growth in the Zloty Stok gold mine [7]. The most closely related named species is *Ensifer adhaerens*, which includes bacterial predators capable of feeding on organisms such as *Micrococcus luteus* [58,61].

### 3.3. Identification of Unique Features of the Ensifer sp. M14 Genome

**Figure 3:**
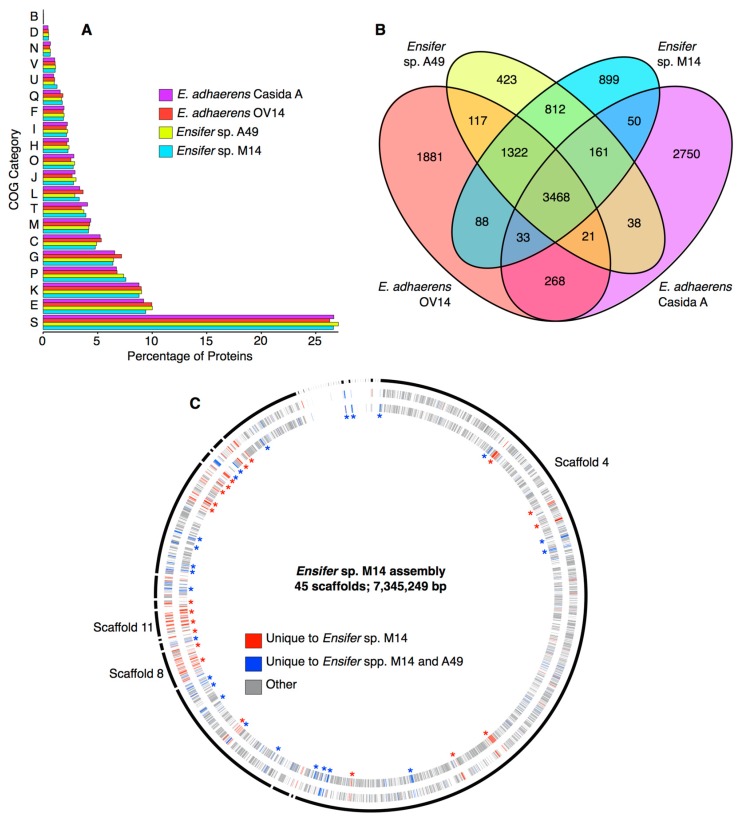
General features of the genome of *Ensifer* sp. M14 and related strains. (**A**) The percentage of proteins encoded by each strain annotated with each COG (Cluster of Orthologous Genes) functional category. COG categories not represented in the proteome are excluded from the graph. COG category definitions are provided in Table S4. (**B**) A Venn diagram indicating the number of genes shared among these four strains, as extracted from the pangenome of the 46 strains shown in Figure 2. (**C**) A circular plot, prepared with Circos version 0.67-7 [62], showing the scaffolds of the *Ensifer* sp. M14 assembly (outer black curved lines) including the plasmids, and the predicted coding sequences on the positive strand (outer ring) and negative strand (inner ring). Scaffolds are drawn proportional to their size, and they are presented in the order they are numbered. Scaffold 4 (chromosome), 8 (pSinB), and 11 (pSinA) are labelled. The coding regions are colored according to their conservation level, with red indicating genes unique to *Ensifer* sp. M14, and yellow indicating species common and unique to *Ensifer* spp. M14 and A49. Some multi-gene loci unique to M14 (red asterisks) or unique to M14 and A49 (blue asterisks) are indicated.

A global, functional analysis of the *Ensifer* sp. M14 proteome was performed using COG categories, and the proteome was compared with closely related species to identify general functional biases. This analysis was performed with the goal of identifying recently acquired genomic islands that may contribute to the adaptation of *Ensifer* sp. M14 to the gold mine environment. When compared with *Ensifer* sp. A49, *E. adhaerens* OV14, and *E. adhaerens* Casida A, no statistically significant biases (pairwise Fisher’s exact tests, *p* > 0.05 in all cases) in COG category abundances were detected in the *Ensifer* sp. M14 proteome (Figure 3A). However, there was a slight, but statistically insignificant (pairwise Fisher’s exact tests, *p* > 0.05), enrichment in inorganic ion transport and metabolism (COG P) in the proteomes of *Ensifer* sp. M14 and *Ensifer* sp. A49 compared to the other two strains (Figure 3A). These results suggest no gross functional changes in the *Ensifer* sp. M14 genome occurred during adaptation to growth in the Zloty Stok gold mine, at least at the general level of COG categories.

Despite the similarity in COG abundances, the *Ensifer* sp. M14 genome contains a large number of unique genes. There are 899 genes found in *Ensifer* sp. M14 but not in *Ensifer* sp. A49, *E. adhaerens* OV14, or *E. adhaerens* Casida A, while an additional 812 are found in M14 and A49, but not OV14 or Casida A (Figure 3B). Of the 899 genes specific to *Ensifer* sp. M14, 656 (9.4% of the genome) were not detected in any of the other 45 *Sinorhizobium*/*Ensifer* strains included in the phylogenetic analysis (Data Sets S1 and S4). Five hundred and ninety of the 656 unique proteins had a blast hit (e-value ≤ 1e^−10^) when queried against the NCBI non-redundant protein database, consistent with the corresponding genes being real genes that were likely acquired from other organisms through horizontal gene transfer (HGT). Mapping the location of the 656 unique genes across the assembly revealed the presence of several putative genomic islands (GIs) likely acquired through recent HGT since the divergence of *Ensifer* sp. M14 from *Ensifer* sp. A49 (Figure 3C, Data Set S4). Scaffolds 11 and 8, which correspond to the pSinA and pSinB plasmids, respectively, were not surprisingly enriched in unique genes, and together account for 217 (33%) of the unique genes. As described in detail elsewhere, these plasmids carry numerous functions associated with arsenic oxidation [10] and heavy metal resistance [10,12]. Of the 439 unique genes spread among the other scaffolds, 309 (70.4%) were annotated as hypothetical genes. Little else of interest was detected among the unique genes (Data Set S4); however, scaffold 36 was predicted to encode a zinc transporting ATPase, and a few genes related to stress resistance or drug resistance were found (discussed later). Overall, these results suggest that essentially all of the recently acquired traits associated with heavy metal resistance, arsenic oxidation, and adaptation to the stressful conditions of the Zloty Stock gold mine are associated with the pSinA and pSinB plasmids.

### 3.4. Metabolism of Ensifer sp. M14

Detailed phenotypic characterization of *Ensifer* sp. M14 was previously reported [7]. To further evaluate (in silico) the metabolic and transport potential of *Ensifer* sp. M14, a draft metabolic reconstruction was prepared encompassing 1491 genes and 1289 gene-associated reactions (Data Set S2). As expected based on the metabolism of related organisms [63], glycolysis in *Ensifer* sp. M14 is predicted to proceed through the Entner–Duodoroff pathway (Figures S3–S5). Growth simulations using Flux Balance Analysis suggested that *Ensifer* sp. M14 has a broad metabolic capacity, with a predicted ability to catabolize 72 carbon sources, including a variety of sugars, sugar alcohols, and organic acids (Table S2). This is consistent with previous work, which found that *Ensifer* sp. M14 could grow on 12 of 16 tested carbon substrates, including glucose, xylose, and lactate [7]. The following paragraphs provide a description of several metabolic capabilities that may be relevant to survival in the stressful environment of the Zloty Stok gold mine, and/or to resistance to elevated arsenic concentrations.

#### 3.4.1. Phosphate Transport

The metabolic reconstruction indicated that *Ensifer* sp. M14 encodes two copies of the PstSCAB-PhoU high-affinity phosphate transporter (*BLJAPNOD_00112* through *BLJAPNOD_00116*; and *BLJAPNOD_05453* through *BLJAPNOD_05457*). Further examination of the *Ensifer* sp. M14 genome additionally revealed two copies of the PhnCDE(T) high-affinity phosphate and phosphonate transport system (*BLJAPNOD_04783* through *BLJAPNOD_04786*; and *BLJAPNOD_05447* through *BLJAPNOD_05450*). Notably, one copy of PstSCAB-PhoU and one copy of PhnCDE(T) were adjacent to the arsenic oxidation gene cluster within pSinA. This led us to explore the presence of phosphate transport systems in other arsenic-oxidizing bacteria (AOB). Using OrthoMCL [37], orthologous proteins were identified among six strains from the family *Rhizobiaceae* (Table S3): these included three AOB (*Ensifer* sp. M14, *A. tumefaciens* 5A, and *Rhizobium* sp. NT-26), as well as three related strains that are not AOB (*E. adhaerens* OV14, *N. galegae* HAMBI 540, and *A. tumefaciens* Ach5). Thirteen proteins were found to be common and specific to the three AOB, which not surprisingly included the arsenic oxidation gene cluster [10]. Notably, included within these 13 proteins were subunits of the PstSCAB-PhoU and PhnCDE(T) transporters. While all six strains encoded orthologous versions of PstSCAB-PhoU and PhnCDE(T), all three AOB encoded additional copies adjacent to their arsenic oxidation loci. Examining the genomes of three additional diverse AOB (*H. arsenicoxydans* ULPAs1, *A. arsenitoxydans* SY8, and *P. stutzeri* TS44) revealed that the first two also contained a second copy of the PstSCAB transporter in close proximity to arsenite related genes.

Based on the above results, we predict that phosphate transport genes are commonly associated with arsenite resistance loci [64]. Arsenates and phosphate are chemical analogs, with the toxicity of arsenic being a result of arsenic replacing phosphate in key biological molecules [65]. Similarly, arsenic competes with phosphate for transport through phosphate transport systems, including the PstSCAB and PhnCDE(T) systems [66,67,68], potentially resulting in phosphate starvation. However, the phosphate periplasmic binding proteins of at least some PstSCAB-PhoU systems, such as from the arsenic-resistant strain *Halomonas* strain GFAJ-1, display a strong preference for binding phosphate over arsenic [68]. Thus, the presence of additional high-affinity phosphate systems in AOB may be a mechanism to increase the rate (and selectivity) of phosphate import, thereby reducing the toxic effects of elevated environmental arsenic concentrations.

#### 3.4.2. Sulfur Metabolism

We evaluated sulfur metabolism by *Ensifer* sp. M14, as sulfur compounds, such as sulfide, can be abundant in gold mines, and the arsenic oxidase enzyme contains an iron-sulfur subunit [64]. *Ensifer* sp. M14 appears to have a variety of mechanisms for sulfate assimilation. Based on the metabolic reconstruction, the genome is predicted to encode multiple sulfate and thiosulfate transporters. It is further predicted to encode several putative thiosulfate sulfurtransferases and a hydrogen sulfide oxidoreductase (*BLJAPNOD_03089*); in contrast, a sulfite oxidoreductase was not identified. Genes *BLJAPNOD_05764* through *BLJAPNOD_05768* may encode for the transport and metabolism of taurine, while *BLJAPNOD_05769* may encode the TauR taurine transcriptional regulator. *Ensifer* sp. M14 is also predicted to encode an alkanesulfonate monoxygenase (*BLJAPNOD_06609*). At least one copy of each of the subunits of the SsuABC alkanesufonate ABC-type transporter are also predicted to be encoded in the genome; however, no locus appeared to contain all three.

#### 3.4.3. One-Carbon Metabolism

*Ensifer* sp. M14 is capable of growing with carbon dioxide or bicarbonate as the sole source of carbon [7], although the underlying metabolic pathway for this capability has not been examined. The metabolic reconstruction identified a putative formamide amidohydrolase (*BLJAPNOD_04973*) and putative formate dehydrogenases (*BLJAPNOD_00952 and BLJAPNOD_03433*), suggestive of the utilization of these one-carbon compounds. No clear evidence for genes associated with methanol or methylamine metabolism were found. However, the mechanism underlying one-carbon metabolism remains unclear. Unlike *S. meliloti* [69], *Ensifer* sp. M14 does not appear to encode the Calvin–Benson–Bassham cycle, nor were we able to identify any of the complete carbon-fixation pathways [70]. However, multiple enzymes potentially involved in the incorporation of bicarbonate were identified. These include putative acetyl-CoA carboxylases (*BLJAPNOD_03269*, *BLJAPNOD_04937*, *BLJAPNOD_04938*), a putative 3-oxopropanoate oxidoreductase (*BLJAPNOD_03990*), putative propanoyl-CoA carboxylases (*BLJAPNOD_06206, BLJAPNOD_06208*), a putative pyruvate carboxylase (*BLJAPNOD_00700*), and a phosphoenolpyruvate carboxylase (*BLJAPNOD_01050*).

#### 3.4.4. Iron Transport and Metabolism

Due to the involvement of iron in arsenic oxidation, the transport and metabolism of this metal was examined. *Ensifer* sp. M14 is predicted to encode several transporters of iron or iron containing compounds. The genes *BLJAPNOD_01755* and *BLJAPNOD_01831* are predicted to encode a ferrous iron (Fe^2+^) permease (EfeU) and a ferrous iron efflux pump (FieF), respectively. Genes *BLJAPNOD_05889* through *BLJAPNOD_05891* may encode a FecBDE ferric dicitrate transporter, while BLJAPNOD_05888 may encode the FecA ferric dicitrate outer membrane receptor protein. The genes *BLJAPNOD_00861* through *BLJAPNOD_00863* may encode a second ferric dicitrate transporter. Additionally, the genes *BLJAPNOD_05777*, *BLJAPNOD_05780*, and *BLJAPNOD_05781* may form an ABC-type transport system for iron or an iron complexes. Moreover, three putative FhuA ferrichrome (iron containing siderophore) transporting outer membrane proteins (*BLJAPNOD_04144*, *BLJAPNOD_04445*, *BLJAPNOD_05778*), and a FcuA ferrichrome receptor (*BLJAPNOD_05962*) are predicted to be encoded in the genome. A putative FepCDG ferric enterobactin transporter (*BLJAPNOD_04147*, *BLJAPNOD_04148*, *BLJAPNOD_04149*) and a PfeA enterobactin receptor (*BLJAPNOD_05560*) are also annotated. Aside from transport, *Ensifer* sp. M14 is predicted to encode a ferric reductase (*BLJAPNOD_01976*–*fhuF*), a ferrous oxidoreductase (*BLJAPNOD_01631*), and a ferric-chelate reductase (*BLJAPNOD_02273*). Additionally, the five gene operon (*BLJAPNOD_05798-BLJAPNOD_05802*) was predicted (using antiSMASH [55]) to encode a siderophore (aerobacin-like) biosynthetic pathway. Finally, the ferric uptake regulator (Fur) is predicted to be encoded by *BLJAPNOD_00930*.

#### 3.4.5. Halotolerance

The *Ensifer* sp. M14 genome was searched for genes relevant to halotolerance as *Ensifer* sp. M14 has been shown to grow in highly saline environments with up to 20 mg L^−1^ NaCl [10]. Examination of the *Ensifer* sp. M14 genome with antiSMASH [55] identified a 13 gene locus (*BLJAPNOD_06859* to *BLJAPNOD_06872*) in which 12 of the genes showed similarity to 12 of the 15 genes of a known salecan biosynthetic cluster. Salecan is a water-soluble β-glucan also produced by the salt tolerant strain *Agrobacterium* sp. ZX09 [71]. Thus, this locus in *Ensifer* sp. M14 may encode for the biosynthesis of salecan, or another carbohydrate, that contributes to halotolerance. Additionally, *Ensifer* sp. M14 is predicted to be capable of synthesizing the compatible solute betaine from choline using the BetA (*BLJAPNOD_01468*, *BLJAPNOD_03726*, *BLJAPNOD_06536*) and BetB (*BLJAPNOD_00678*, *BLJAPNOD_03725*, *BLJAPNOD_05671*) pathway, as well as from choline-O-sulfate with BetC (*BLJAPNOD_02271*, *BLJAPNOD_03724*). The genome is further predicted to encode numerous proteins related to glycine betaine and proline betaine transport. Finally, as previously reported [10], pSinA encodes a putative NhaA pH-dependent sodium/proton antiporter (*BLJAPNOD_05431*), which may contribute to adaptation to high salinity [72].

#### 3.4.6. Heavy Metal Resistance

*Ensifer* sp. M14 displays high resistance to numerous heavy metals [7]. Previous work identified eight modules related to heavy metal resistance on the pSinB replicon of *Ensifer* sp. M14 [12]. These modules were involved in resistance to arsenic, cadmium, cobalt, copper, iron, mercury, nickel, silver, and zinc [12]. Additionally, pSinA contains a locus involved in resistance to cadmium, zinc, cobalt, and mercury [10]. Our analyses reported above suggested that the majority, if not all, genes relevant to adaptation to the heavy metal-rich environment in the Zloty Stok gold mine are located on the pSinA and pSinB plasmids [10,12].

### 3.5. Biosafety Considerations of Ensifer sp. M14

The *Sinorhizobium*/*Ensifer* group of bacteria contain numerous plant symbionts and other biotechnologically relevant strains, but it lacks known pathogens. Considering this, and the observation that none of the genomic islands detected in *Ensifer* sp. M14 appear to be pathogenicity islands, it is unlikely that *Ensifer* sp. M14 is pathogenic. Therefore, the environmental release of *Ensifer* sp. M14 is not expected to pose a biosafety risk from that perspective. Additionally, analysis of the secondary metabolism of *Ensifer* sp. M14 with antiSMASH [55] did not identify antibiotic synthesis loci. However, *Ensifer* sp. M14 may carry several antimicrobial resistance (AMR) genes. The analysis applying the RGI analyzer revealed the presence of 12 putative antibiotic resistance genes/gene clusters (Table 2). It is worth mentioning that the best hits were found for four *acrAB(-TolC)* modules encoding resistance-nodulation-cell division (RND) type multidrug efflux systems, while the remaining eight genes were much more divergent compared with the reference proteins (they were detected only when applying the LOOSE algorithm of the RGI analyzer). This may suggest that these hits are accidental, and that the identified genes are not truly AMR genes, or that these are novel, emergent threats and more distant homologs of known reference genes.

Previous analyses revealed that the closely related organism *E. adhaerens* OV14 displays resistance to numerous antibiotics, including, among others, ampicillin, spectinomycin, kanamycin, and carbenicillin [73]. Therefore, to check whether the predicted antibiotic resistance genes truly associated with antibiotic resistance in *Ensifer* sp. M14, the MICs of 11 antibiotics were determined using Etests. Results from the Etests showed that *Ensifer* sp. M14 is resistant to ampicillin (MIC: 12.0 mg L^−1^), chloramphenicol (MIC: 8.0 mg L^−1^), and rifampicin (MIC 4.0 mg L^−1^), while it is susceptible to cefixime, cefotaxime, ceftriaxone, ciprofloxacin, gentamicin, moxifloxacin, and trimethoprim. In the case of tetracycline, the MIC values fluctuated around the threshold for classification as resistant (1–4 mg L^−1^); hence, precise interpretation of this result is not possible. Resistance to antibiotics belonging to the penams, phenicols, and rifamicyns families may be explained by the presence of efflux pumps belonging to the RND family. These multidrug resistance systems are highly prevalent in Gram-negative bacteria, and play an important role in resistance to various types of stress factors, including antibiotics [74]. It is also worth mentioning, that environmental isolates of *Alphaproteobacteria* usually possess several copies of genetic modules encoding RND type multidrug efflux systems, which may be linked with their adaptation to the heterogeneity of the soil habitat [75,76]. Therefore, we think that the environmental release of *Ensfier* sp. M14 is unlikely to pose a biosafety risk.

### 3.6. Development of a Pilot-Scale Installation for Arsenic Bioremediation

The genomic analyses suggested that *Ensifer* sp. M14 contains several genetic features that may allow it to be successfully used in environmental bioremediation applications. In addition, previous experimental studies demonstrated that this strain can efficiently transform As(III) into As(V) (24 h of residence time was sufficient to oxidize 5 mg L^−1^ of As(III) in the laboratory) [9]. We therefore attempted to prepare an installation for environmental bioremediation of arsenic contaminated water involving *Ensifer* sp. M14. The purification of arsenic contaminated waters constitutes a serious environmental challenge, as most of the available chemical and physical methods are dedicated to the selective removal of As(V), and are inefficient with regard to As(III). Thus, the aim of the microbiological module of the installation was to harness the arsenite oxidation capabilities of *Ensifer* sp. M14 to ensure efficient oxidation of As(III) to facilitate its subsequent removal. We reasoned that combining a biological approach with an appropriate physicochemical process (i.e., adsorption) could overcome the constraints and reservations of the conventional methods dedicated to the removal of arsenic from contaminated waters [77,78].

### 3.7. The Activity and Characterization of the Microbiological Module of the Pilot-Scale Installation

In our preliminary study [9], we observed that efficient functioning of the laboratory-scale installation required a high density of *Ensifer* sp. M14 (OD_600_ between 0.1 and 0.2). This is in part because the quantity of *Ensifer* sp. M14 usually decreases quite intensively during the first hours/days of continuous culturing in the bioreactor [9]. Although appropriate growth conditions and length of residence time during continuous cultures were previously determined [9], the move from the laboratory-scale to pilot-scale installation meant it was necessary to re-evaluate them. In particular, replacement of the synthetic medium by natural arsenic contaminated water, as well as increasing the scale of application, may result in a deceleration of bacterial growth and a decrease in the efficiency of the biooxidation processes [79].

#### 3.7.1. Microbial Growth and Efficiency of Arsenic Biooxidation in the Bioreactor

**Figure 4:**
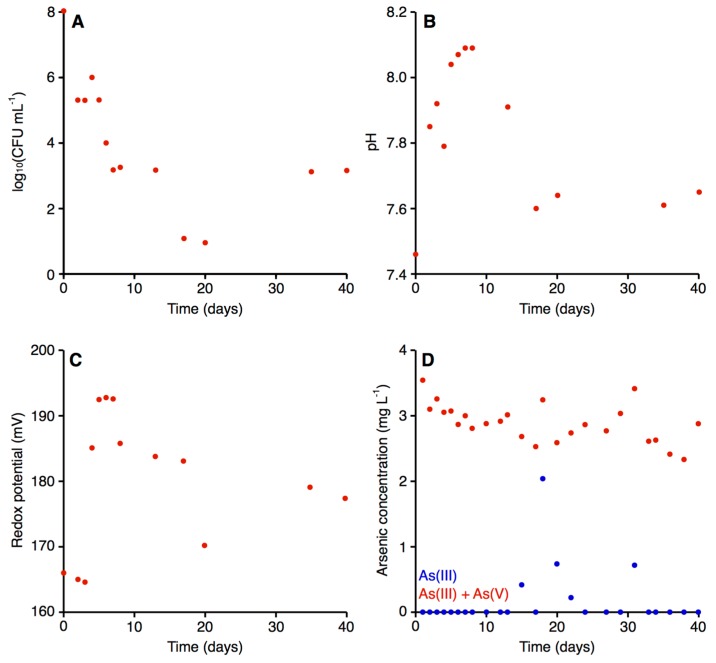
Parameters of the water in the bioreactor of the microbiological module. The graphs display (**A**) the quantity of bacteria, (**B**) the pH of the water, (**C**) the redox potential of the water, and (**D**) the concentration of As(III) (blue) and total arsenic (red) in the water.

Using the start-up procedures described in the Materials and Methods, the initial quantity of bacteria in the bioreactor after yeast extract augmentation was about 10^8^ CFU mL^−1^ (Figure 4). The value was almost nine orders of magnitude higher compared to raw arsenic-contaminated water, where the CFU mL^−1^ (when plated on Luria-Bertani agar medium) was about 10^0^.

As expected based on our preliminary study [9], the density of bacteria decreased systematically during the first few days of operation, reaching a density on the magnitude of 10^3^ CFU mL^−1^ on day seven (Figure 4A). After this point, the density of bacteria largely stabilized, with the exception of a few days (days 17–20), when an ~100-fold drop in bacterial density was observed (Figure 4A). A bacterial concentration of 10^3^ CFU mL^−1^ in the bioreactor generally appeared sufficient for efficient biooxidation of the arsenite in the contaminated water, as there was generally little to no arsenite detected in the water following passage through the bioreactor (Figure 4D). The exceptions were five of the nine measurements taken between days 15 and 31, inclusive, when arsenite accounted for up to 62.86% of the total arsenic concentration; this corresponded with the drop in the density of bacteria within the bioreactor (Figure 4A). Thus, the low arsenite concentration throughout the majority of the experiment suggests that the microbiological module efficiently converted the As(III) to As(V).

Recently, Tardy et al. [52] showed that efficient arsenite biooxidation in environmental samples of water at 20 °C occurred after eight days of culture (batch experiment), and the quantity of bacteria at the end of their experiment was about 10^5^ CFU mL^−1^. On the other hand, Kamde et al. [80] reported that arsenic removal was most intensive when the quantity of bacteria was about 28 CFU mL^−1^ (batch cultures with the use on synthetic medium). The higher quantity of bacteria in the abovementioned papers in comparison with our study is presumably related to differences in culture conditions (various media and/or culturing methods).

Our data (Figure 4A,D) is also consistent with a relationship between the quantity of *Ensifer* sp. M14 and the efficiency of arsenic biooxidation, as were our preliminary experiments in batch cultures (data not shown). Indeed, many studies have observed a positive correlation between the density of bacteria and the rate of metal metabolism or biotransformation for arsenic compounds and other elements [80,81,82].

#### 3.7.2. Physical and Chemical Characterization of the Bioreactor

Previous studies have observed that there is a relationship between pH and redox potential with the arsenite/arsenate ratio; arsenites are the predominant form in reducing conditions and lower pH values, as the concentration of the arsenate form increases, both pH and redox potential also increase [83,84]. We therefore evaluated the pH and the redox potential in the treated water. For both parameters, the biological treatment had a small but noticeable effect. In the raw water, the pH and the redox potential were 7.48 and 170.90 mV, respectively [56]. In the case of the pH, the value in the bioreactor systematically increased up to the eighth day, with the treated water reaching a pH of 8.09 (Figure 4B). The pH returned to 7.60 by day 17, following which the pH stabilized in the range of 7.60 to 7.65 until the end of the experiment (day 40). In general, the redox potential remained relatively stable (Figure 4C). For the first three days, a value around 155.00 mV was observed, following which the redox potential increased and stabilized (with a slight, gradual decrease) within a range from 177.00 mV and 193.00 mV, with the exception of day 20. Water for human consumption is expected to have a pH in the range of 6.50–9.00 [85] and a redox potential between 100 and 300.00 mV [86]. Thus, both the pH and the redox potential of the water treated with our installation fell within the acceptable range for drinking water.

### 3.8. Effectiveness of the Adsorption Module of the Pilot-Scale Installation

Granulated bog iron ores are characterized by high arsenic adsorption capacity (up to 5.72 mg kg^−1^, depending on the adsorbate concentration), short residence time (20 min) [56], they display high chemical stability, and they are resistant to bioweathering processes [87]. These properties allow this material to function as an effective adsorbent for removal of arsenics from contaminated water in both passive and active remediation systems, as demonstrated in our earlier work [56]. Here, we have coupled the use of granulated bog iron ores as an input to the adsorption module as well as the microbiological module described above, as a way to ensure the efficient conversion of As(III) to As(V) by *Ensifer* sp. M14, followed by the removal of As(V) by the bog iron ores. The pre-conversion of As(III) to As(V) is important as bog iron ores saturated with As(V) display higher chemical stability than bog iron ores saturated with As(III) [87].

**Figure 5:**
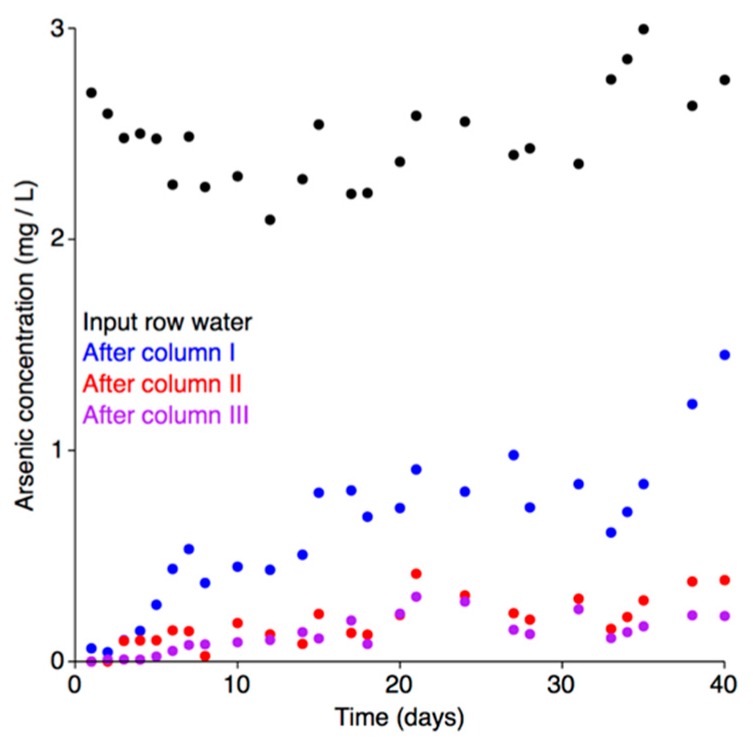
Arsenic adsorption breakthrough curves. The arsenic adsorption breakthrough curves for each column of the adsorption module are shown. Total arsenic concentrations in the raw water (black), and after column I (blue), column II (red), and column III (purple) are shown.

Treatment of the arsenic contaminated water with the pilot-scale installation led to a dramatic decrease in arsenic concentrations, going from 2400 μg L^−1^ in the raw water to less than 10 μg L^−1^ (Figure 5). Analysis of the breakthrough curves for each of the adsorption columns indicated that the adsorbent in none of the columns reached equilibrium saturation during the 40-day experiment (Figure 5). Equilibrium saturation is herein defined as the maximum adsorption capacity (full saturation) of the adsorbent at a given concentration of the adsorbate; i.e., when the arsenic concentration in the input and output water is equal. Upon reaching equilibrium saturation, the adsorbent would be completely consumed and unable to further remove arsenic from the water, and it would therefore require regeneration or replacement. As the total arsenic concentration in water after each column was lower than the water entering the column, none of the columns reached equilibrium saturation. Thus, under the tested environmental conditions, the pilot-scale installation is expected to have been able to effectively continue the bioremediation process for much longer than the 40 days of the experiment (during which, 8 m^3^ of water flowed through the system).

In Poland, the Regulation of the Polish Ministry of the Environment [88] currently sets the upper limit for arsenic contamination in water for use in technological purposes at 100 μg L^−1^. In the experiment reported here, the arsenic concentration in the treated water remained below 100 μg L^−1^ for the first ten days of the experiment (Figure 5), and never exceeded 220 μg L^−1^ during the 40 day test. Thus, at least the first 2.0 m^3^ of water treated by pilot-scale installation was below the Polish limit for use in technological purposes. However, if pooling the treated water (and thus averaging the arsenic concentration), it is likely that the cumulative concentration of arsenic in the 4.0 m^3^ of water treated over the first 20 days remained below the limit.

The local adsorption capacity of the adsorbent varied between the columns and depended on the arsenic concentration of the inflowing water. The adsorbent from the first column was characterized by the highest adsorption capacity, which was 0.500 mg kg^−1^. Adsorbent placed in the second and third columns had lower adsorption capacities of 0.031 and 0.021 mg kg^−1^, respectively. Likely, these differences are due to the later columns adsorbing less arsenic and being farther from reaching equilibrium saturation. The adsorption capacities recorded in the current study were significantly lower than those described in our previous work, presumably due to the adsorbent not reaching equilibrium saturation [56].

## 4. Conclusions

Here, we reported the draft genome sequence of *Ensifer* sp. M14 in order to gain insights into the genomic adaptation of this organism to the stressful environment of the abandoned Zloty Stok gold mine from which it was isolated. In addition, we were interested in the genetic basis of the strains arsenic oxidation and resistance capabilities, resistance to arsenic and other heavy metals, and the biosafety of the strain for use in biotechnological applications. The results revealed hundreds of genes present in *Ensifer* sp. M14 that are not found in related species, and these genes are often colocalized in genomic islands. However, the majority of these genes encoded hypothetical proteins of unknown function. Based on the genome sequence, it appears that the majority of the genes have been acquired to deal with the hostile environment of the Zloty Stok gold mine, i.e., conferring resistance to heavy metals, and enabling arsenic oxidation, are located on the self-transmissible pSinA and pSinB megaplasmids. Additionally, analysis of the *Ensifer* sp. M14 genome suggested that this strain should be safe for use in biotechnology and bioremediation. However, it was noted that several putative antibiotic resistance genes are present in the genome, as is also true for the related strain *Ensifer adhaerens* OV14 that is used in biotechnological applications [89]. This property of *Ensifer* sp. M14 should be kept in mind during its application in order to limit the spread of antimicrobial resistance. The results of these genomic analyses provide hints into the genetic potential of *Ensifer* sp. M14. They will help focus future experimental research aimed at further characterizing the biology of this organism, and may contribute to the development of procedures for large-scale cultivation of this strain.

This study also reports the construction and validation of a pilot-scale installation designed for the remediation of arsenic contaminated waters. This novel installation couples a microbiological module, based on the arsenic oxidation abilities of *Ensifer* sp. M14, with an adsorption module, based on the use of granulated bog iron ores. The underlying principle is to use *Ensifer* sp. M14 to efficiently oxidize the As(III) ions to As(V), followed by the removal of the As(V) through adsorption by the bog iron ores. Characterization of the arsenic contaminated water following passage through the microbiological module generally revealed little to no As(III), consistent with the *Ensifer* sp. M14 generally ensuring effective conversion of As(III) to As(V). Additionally, a dramatic decrease (from 10-fold to greater than 250-fold) in the arsenic concentration of the water was observed following passage of this water through the adsorption module. These results therefore confirm the effectiveness of the tested installation for the remediation of arsenic contaminated waters, which pose risks to both the environmental and human health. Future work will be aimed at further developing and optimizing this system, which could involve, for example, the addition of beads to the reactor containing *Ensifer* sp. M14 biofilms.

## Figures and Tables

**Table 1:** Features of the *Ensifer* sp. M14 genome assembly.

Length	7,345,249 bp
G + C content	61.47%
CDS	6874
rRNA	3
tRNA	53
Miscellaneous RNA	33
Scaffolds	45
Scaffold N50 (L50)	4400,487 (1)
CDS with COG terms *^,†^	64.00%
CDS with GO terms *	28.70%
CDS with KEGG pathway terms *	35.50%
CDS with eggNOG annotations *^,¥^	80.50%
CDS with no similarity *	9.40%

* As determined using eggnog-mapper [24]. Those genes not returned in the eggNOG-mapper output were said to have no similarity; ^†^ Excluding those annotated with COG category S (unknown function); ^¥^ Excluding those annotated as protein/domain of unknown/uncharacterized function. CDS (Coding Sequences); COG (Cluster of Orthologous Genes); KEGG (Kyoto Encyclopedia of Genes and Genomes); GO (Gene Ontology).

**Table 2:** Putative antimicrobial resistance genes found in the *Ensifer* sp. M14 genome.

Scaffold	Gene ID	CARD Database Hit	Predicted Resistance to	Tested Antibiotics
**Scaffold_4**	***BLJAPNOD_00187-***	***acrAB***	**Fluoroquinolone**	**CIP (S); MXF (S)**
	***BLJAPNOD_00188***		**Tetracyclines**	**TE (S/R)**
Scaffold_4	*BLJAPNOD_00458*	*cmlA/floR*	Chloramphenicol	C (R)
**Scaffold_4**	***BLJAPNOD_00485-***	***acrAB-TolC***	**Tetracyclines**	**TE (S/R)**
	***BLJAPNOD_00487***		**Cephalosporins**	**CFM (S); CRO (S); CTX (S)**
			**Penams**	**AMP (R)**
			**Phenicols**	**C (R)**
			**Rifamycins**	**RD (R)**
			**Fluoroquinolones**	**CIP (S); MXF (S)**
Scaffold_4	*BLJAPNOD_00960*	*aph(3′)-IIa*	Aminoglycosides	CN (S)
Scaffold_4	*BLJAPNOD_01284*	*adeF*	Fluoroquinolones	CIP (S); MXF (S)
			Tetracyclines	TE (S/R)
Scaffold_4	*BLJAPNOD_02256*	*bla_OXA_*	Cephalosporins	CFM (S); CRO (S); CTX (S)
			Penams	AMP (R)
Scaffold_4	*BLJAPNOD_02798*	*aph(6)-Ic*	Aminoglycosides	CN (S)
Scaffold_7	*BLJAPNOD_04982*	*aph(3′′)-Ib*	Aminoglycosides	CN (S)
**Scaffold_8**	***BLJAPNOD_05149-***	***acrAB-TolC***	**Tetracyclines**	**TE (S/R)**
	***BLJAPNOD_05151***		**Cephalosporins**	**CFM (S); CRO (S); CTX (S)**
			**Penams**	**AMP (R)**
			**Phenicols**	**C (R)**
			**Rifamycins**	**RD (R)**
			**Fluoroquinolones**	**CIP (S); MXF (S)**
**Scaffold_14**	***BLJAPNOD_05841-***	***acrAB***	**Fluoroquinolone**	**CIP (S); MXF (S)**
	***BLJAPNOD_05842***		**Tetracyclines**	**TE (S/R)**
Scaffold_17	*BLJAPNOD_06442*	*dfrA12*	Trimethoprim	TM (S)
Scaffold_18	*BLJAPNOD_06615*	*aph(6)-Ic*	Aminoglycosides	CN (S)

The most significant hits, defined with the usage of the STRICT algorithm of the RGI analyzer, are bolded. Abbreviations: AMP—ampicilin; C—chloramphenicol; CN—gentamicin; CFM—cefixime; CTX—cefotaxime, CRO—ceftriaxone; CIP—ciprofloxacin; TE—tetracycline; TM—trimethoprim; MXF—moxifloxacin; RIF—rifampicin; R—resistant; S—susceptibility; S/R—inability of interpretation of the result (threshold value).

## Supplementary Materials

The following are available online at http://www.mdpi.com/2073-4425/9/8/379/s1. Table S1: Accession numbers for all genomes used in this work, Table S2: In silico test of the metabolic capacity of *Ensifer* sp. M14, Table S3: Orthologous groupings of six *Rhizobiaceae* strains, including three AOB and three strains that are not AOB, Table S4: COG category descriptions, Figure S1: Average nucleotide identity matrix. A matrix of the two-way ANI values for 46 *Sinorhizobium*/*Ensifer* strains is shown. Clustering was performed along both axes using hierarchical clustering with Pearson distance and average linkage, Figure S2: Average amino acid identity matrix. A matrix of the two-way AAI values for 46 *Sinorhizobium*/*Ensifer* strains is shown. Clustering was performed along both axes using hierarchical clustering with Pearson distance and average linkage, Figure S3: Entner–Duodoroff pathway and the pentose phosphate pathway. A modified version of the KEGG pathway map ko00030 [90] displaying the Entner–Duodoroff pathway and the pentose phosphate pathway is shown. Reactions encoded by the *Ensifer* sp. M14 genome are colored green; those in white are missing. The figure was prepared using the KAAS webserver [91] using BLAST search with the bi-directional best hit assignment method, and with the default organism list for ‘prokaryotes’ plus *Sinorhizobium meliloti* Rm1021, Figure S4: Gluconeogenesis. A modified version of the KEGG pathway map ko00010 [90] displaying the pathway for gluconeogenesis is shown. Reactions encoded by the *Ensifer* sp. M14 genome are colored green; those in white are missing. The figure was prepared using the KAAS webserver [91] using BLAST search with the bi-directional best hit assignment method, and with the default organism list for ‘prokaryotes’ plus *Sinorhizobium meliloti* Rm1021, Figure S5: Tricarboxylic acid cycle. A modified version of the KEGG pathway map ko00020 [90] displaying the tricarboxylic acid (TCA) cycle is shown. Reactions encoded by the *Ensifer* sp. M14 genome are colored green; those in white are missing. The figure was prepared using the KAAS webserver [91] using BLAST search with the bi-directional best hit assignment method, and with the default organism list for ‘prokaryotes’ plus *Sinorhizobium meliloti* Rm1021, Data Set S1: *Sinorhizobium*/*Ensifer* gene presence and absence. This file contains the gene presence/absence output data from Roary for the pangenome analysis of 46 *Sinorhizobium*/*Ensifer* strains. Details on the information provided in the file is available at: https://sanger-pathogens.github.io/Roary/, Data Set S2: Metabolic reconstruction of *Ensifer* sp. M14. This archive contains the expanded, draft metabolic reconstruction of *Ensifer* sp. M14. The reconstruction is provided in COBRA format as a MATLAB file, as well as in a table within an Excel workbook. A readme file is included to explain the contents, Data Set S3: PhiSpy phage prediction. This file contains the PhiSpy phage prediction output for all *Ensifer* sp. M14 genes, as well as separate sheets for each of the putative prophage loci. Details on the information provided in the file is available at: https://github.com/linsalrob/PhiSpy, Data Set S4: Functional annotation of the *Ensifer* sp. M14 genome. This file contains the genome annotation and the eggNOG-mapper output for three sets of genes: (i) all genes in the *Ensifer* sp. M14 genome; (ii) all genes unique to the *Ensifer* sp. M14 genome; and (iii) all genes unique and common to the *Ensifer* sp. M14 and A49 genomes. Details on the eggNOG-mapper output provided in the file is available at: https://github.com/jhcepas/eggnog-mapper/wiki/Results-Interpretation.
