# Supplementary material for: Genomic and Biotechnological Characterization of the Heavy-Metal Resistant, Arsenic-Oxidizing Bacterium Ensifer sp. M14
Source: Genes (Basel). 2018 Jul 27;9(8):379. doi: 10.3390/genes9080379 (PMC6115938; doi:10.3390/genes9080379)
Supplement: Supplementary file 1 [file genes-09-00379-s001.zip › genes-323031-supplementary_GD/Figure_S1.pdf]

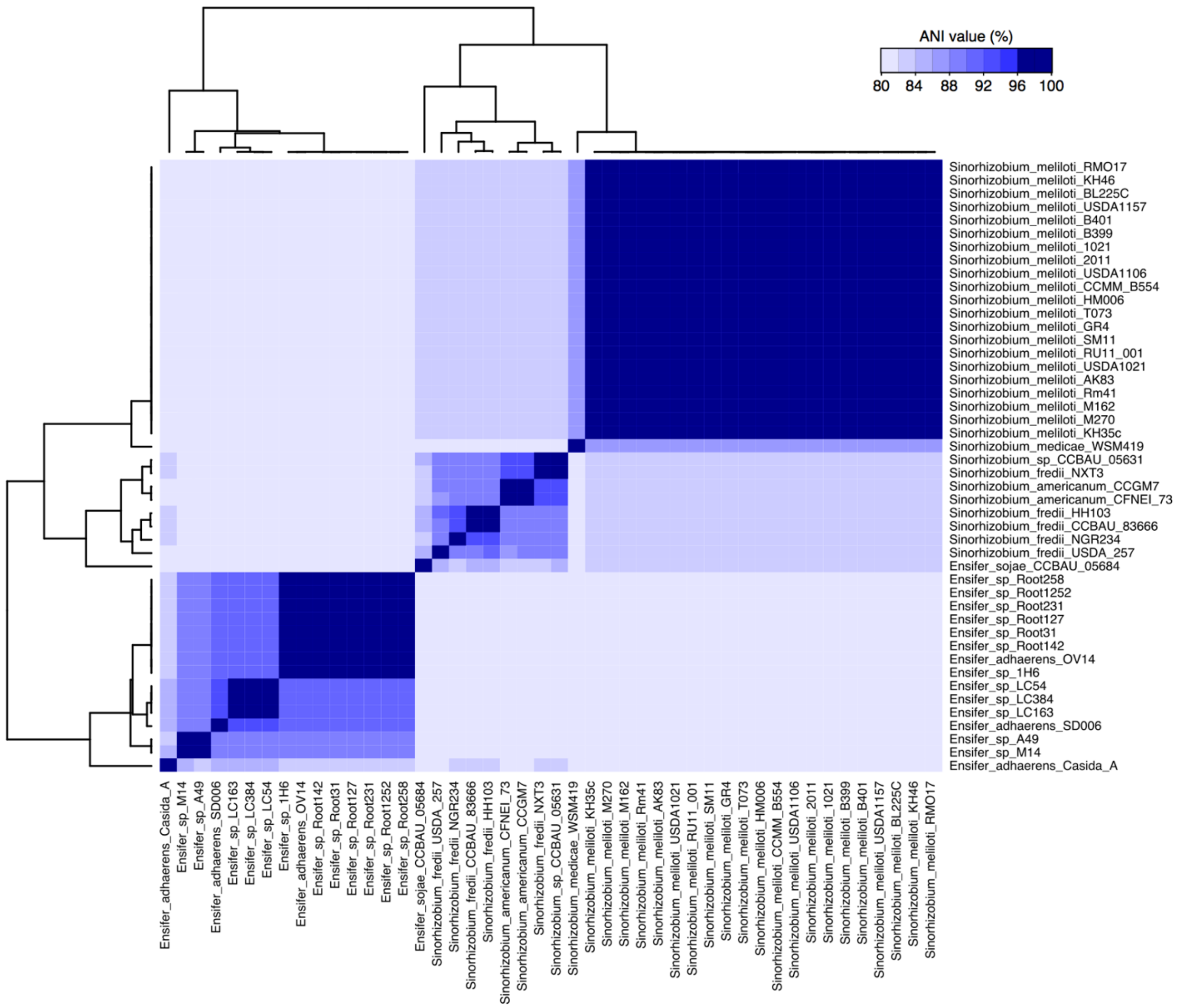


**Figure S1: Average nucleotide identity matrix.** A matrix of the two-way ANI values for 46 *Sinorhizobium*/*Ensifer* strains is shown. Clustering was performed along both axes using hierarchical clustering with Pearson distance and average linkage.
