# Supplementary material for: Genomic and Biotechnological Characterization of the Heavy-Metal Resistant, Arsenic-Oxidizing Bacterium Ensifer sp. M14
Source: Genes (Basel). 2018 Jul 27;9(8):379. doi: 10.3390/genes9080379 (PMC6115938; doi:10.3390/genes9080379)
Supplement: Supplementary file 1 [file genes-09-00379-s001.zip › genes-323031-supplementary_GD/Figure_S4.pdf]

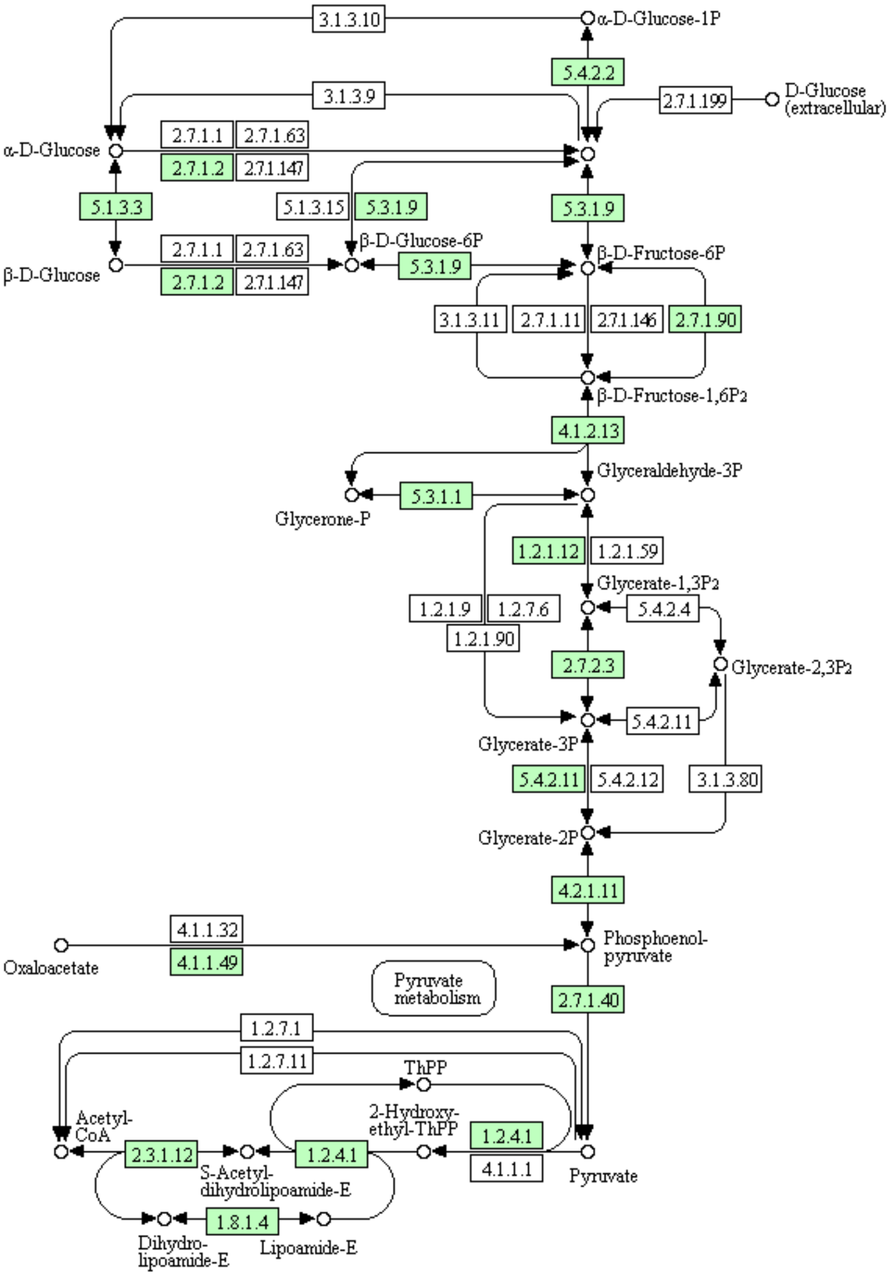

Figure S4: Gluconeogenesis. A modified version of the KEGG pathway map ko00010 [1] displaying the pathway for gluconeogenesis is shown. Reactions encoded by the *Ensifer* sp. M14 genome are colored green; those in white are missing. The figure was prepared using the KAAS webserver [2] using BLAST search with the bi-directional best hit assignment method, and with the default organism list for ‘prokaryotes’ plus *Sinorhizobium meliloti* Rm1021.

1. Kanehisa, M.; Sato, Y.; Kawashima, M.; Furumichi, M.; Tanabe, M. KEGG as a reference resource for gene and protein annotation. *Nucleic Acids Res.* **2016**, *44*, D457–D462.
2. Moriya, Y.; Itoh, M.; Okuda, S.; Yoshizawa, A. C.; Kanehisa, M. KAAS: an automatic genome annotation and pathway reconstruction server. *Nucleic Acids Res.* **2007**, *35*, W182–5.
