# Supplementary material for: Genomic and Biotechnological Characterization of the Heavy-Metal Resistant, Arsenic-Oxidizing Bacterium Ensifer sp. M14
Source: Genes (Basel). 2018 Jul 27;9(8):379. doi: 10.3390/genes9080379 (PMC6115938; doi:10.3390/genes9080379)
Supplement: Supplementary file 1 [file genes-09-00379-s001.zip › genes-323031-supplementary_GD/Table_S4.pdf]

Table S3. COG category descriptions.

COG Category	Description
B	Chromatin structure and dynamics
C	Energy production and conversion
D	Cell cycle control, cell division, and chromosome partitioning
E	Amino acid transport and metabolism
F	Nucleotide transport and metabolism
G	Carbohydrate transport and metabolism
H	Coenzyme transport and metabolism
I	Lipid transport and metabolism
J	Translation, ribosomal structure and biogenesis
K	Transcription
L	Replication, recombination, and repair
M	Cell wall, membrane, envelope biogenesis
N	Cell motility
O	Posttranslational modification, protein turnover, and chaperones
P	Inorganic ion transport and metabolism
Q	Secondary metabolite biosynthesis, transport, and catabolism
S	Unknown function
Y	Signal transduction mechanisms
U	Intracellular trafficking, secretion, and vesicular transport
V	Defense mechanisms
